# Ozone Treatment in the Management of Chemotherapy-Induced Peripheral Neuropathy: A Review of Rationale and Research Directions

**DOI:** 10.3390/cancers17142278

**Published:** 2025-07-08

**Authors:** Bernardino Clavo, Angeles Cánovas-Molina, Mario Federico, Gregorio Martínez-Sánchez, Gretel Benítez, Saray Galván, Yolanda Ramallo-Fariña, Himar Fabelo, Sara Cazorla-Rivero, Elba Lago-Moreno, Carla Antonilli, Juan A. Díaz-Garrido, Ignacio J. Jorge, Gustavo Marrero-Callico, Delvys Rodríguez-Abreu, Francisco Rodríguez-Esparragón

**Affiliations:** 1 Research Unit, Hospital Universitario de Gran Canaria Dr. Negrín, 35019 Las Palmas de Gran Canaria, Spain; canovaspi@hotmail.com (A.C.-M.); mariofedericos@yahoo.it (M.F.); hfabelo@iuma.ulpgc.es (H.F.); scazorla@ull.edu.es (S.C.-R.); elbiskara2020@gmail.com (E.L.-M.); 2 Chronic Pain Unit, Hospital Universitario de Gran Canaria Dr. Negrín, 35019 Las Palmas de Gran Canaria, Spain; ijja36@gmail.com; 3 Radiation Oncology Department, Hospital Universitario de Gran Canaria Dr. Negrín, 35019 Las Palmas de Gran Canaria, Spain; 4 Fundación Canaria Instituto de Investigación Sanitaria de Canarias (FIISC), 35019 Las Palmas de Gran Canaria, Spain; yolanda.ramallofarina@sescs.es (Y.R.-F.); juanantdiaz@hotmail.com (J.A.D.-G.); 5 Molecular and Translational Pharmacology Group, University Institute for Research in Biomedicine and Health (iUIBS), University of Las Palmas de Gran Canaria, 35016 Las Palmas de Gran Canaria, Spain; 6 Instituto Universitario de Enfermedades Tropicales y Salud Pública de Canarias de la Universidad de La Laguna, 38296 Santa Cruz de Tenerife, Spain; 7 CIBER de Enfermedades Infecciosas, Instituto de Salud Carlos III, 28029 Madrid, Spain; 8 Spanish Group of Clinical Research in Radiation Oncology (GICOR), 28290 Madrid, Spain; 9 Scientific Advisor, Freelance, 60126 Ancona, Italy; gregorcuba@yahoo.it; 10 Medical Oncology Department, Complejo Hospitalario Universitario Insular Materno-Infantil de Gran Canaria, 35016 Las Palmas de Gran Canaria, Spain; gretelbenitez@yahoo.es (G.B.); drodabr@gobiernodecanarias.org (D.R.-A.); 11 Medical Oncology Department, Hospital Universitario de Gran Canaria Dr. Negrín, 35019 Las Palmas de Gran Canaria, Spain; saraygalrui@gmail.com (S.G.); carlaan@hotmail.com (C.A.); 12 Network for Research on Chronicity, Primary Care, and Health Promotion (RICAPPS), 08007 Barcelona, Spain; 13 Servicio de Evaluación y Planificación del Servicio Canario de Salud (SESCS), 38109 Santa Cruz de Tenerife, Spain; 14 Institute for Applied Microelectronics (IUMA), University of Las Palmas de Gran Canaria (ULPGC), 35017 Las Palmas de Gran Canaria, Spain; gustavo@iuma.ulpgc.es; 15 Department of Psychiatry, Hospital Universitario de Gran Canaria Dr. Negrín, 35019 Las Palmas de Gran Canaria, Spain; 16 Facultad de Psicología, Universidad Fernando Pessoa Canarias, 35450 Las Palmas de Gran Canaria, Spain

**Keywords:** survivors, chemotherapy-induced peripheral neuropathy, grade of toxicity, neuropathic pain, Nrf2, numbness and tingling, oxidative stress modulation, ozone treatment, quality of life, side effects

## Abstract

Background: Chemotherapy-induced peripheral neuropathy (CIPN) is a common side effect of chemotherapy. CIPN can lead to a dose reduction and/or the interruption of chemotherapy, limiting its effectiveness, while chronic CIPN decreases patients’ quality of life. Improvements in cancer treatment and patients’ survival have increased the number of patients living with CIPN. The only evidence-based treatment for CIPN-related pain, duloxetine, provides only modest clinical benefit, and there is no effective clinical management option for numbness and tingling. Several experimental studies and clinical reports suggest that adjuvant ozone treatment may be beneficial in managing CIPN. Methods: This narrative review aims to provide an overview of current knowledge regarding CIPN and ozone therapy. Specifically, it summarizes experimental studies (18) and clinical reports (27) published between 1995 and 2025 that offer preliminary evidence supporting the potential role of ozone treatment in managing CIPN, highlighting the need for ongoing randomized clinical trials to establish its efficacy. Additionally, this review highlights existing gaps in the literature and proposes directions for future research. Results: The hypothesized mechanisms of action and experimental findings suggest that ozone therapy may be a valuable intervention for CIPN, a concept supported by preliminary clinical observations. Conclusions: Clinically relevant approaches for established CIPN are currently unavailable. While preliminary data suggest a potential role of ozone therapy, clinical evidence remains limited. Further high-quality randomized controlled trials are needed to confirm its efficacy and safety in this context; several trials are currently ongoing.

## Simple Summary

Chemotherapy-induced peripheral neuropathy (CIPN) is a common side effect of chemotherapy. CIPN can lead to a dose reduction and/or the interruption of chemotherapy, limiting its effectiveness, while chronic CIPN decreases patients’ quality of life. Unfortunately, the only evidence-based treatment for CIPN-related pain, duloxetine, provides only a modest clinical benefit, and there is no effective clinical management option for numbness and tingling. This narrative review summarizes the mechanisms underlying CIPN and the effects of ozone. It also summarizes experimental studies (18) and clinical reports (27) published between 1995 and 2025 that offer preliminary evidence supporting the potential role of ozone treatment in managing CIPN and highlight the need for ongoing randomized clinical trials to establish its efficacy.

## 1. Introduction

Chemotherapy-induced peripheral neuropathy (CIPN) is a debilitating dose-limiting side effect of several commonly used chemotherapeutic agents, including platinum compounds, taxanes, vinca alkaloids, and proteasome inhibitors. CIPN manifests with a range of sensory, motor, and autonomic symptoms, including pain, allodynia, hypoesthesia, numbness, tingling, and impaired balance, with a significant impact on patients’ quality of life [1,2,3,4]. The prevalence of CIPN varies from 19% to 100%, depending on the specific chemotherapeutic regimen, dosage, treatment duration, and patient risk factors. However, it can affect up to 70–100% of patients receiving taxanes or platinum-based chemotherapy [5].

The pathophysiology of CIPN is complex, involving multiple mechanisms including oxidative stress, inflammation, and the disruption of neuronal structure and function [5,6,7]. These mechanisms are briefly mentioned here to provide context and are discussed in detail in Section 2.

Current treatment strategies for CIPN are mainly limited to symptomatic relief, with duloxetine being the only agent recommended by major guidelines, with moderate evidence of its efficacy, and only for pain; in particular, no effective therapies exist for numbness and tingling, which are the most frequent and distressing symptoms [1,6,7]. Moreover, existing pharmacological options such as gabapentin and pregabalin often provide only partial benefit, have significant side effects, and do not target the underlying nerve damage. This highlights a substantial unmet need for effective, safe, and disease-modifying treatments for CIPN, as emphasized by the American Society of Clinical Oncology (ASCO) [8].

Given these limitations and the suboptimal benefits from most existing interventions [1,9,10], there is a growing interest in novel approaches that target the underlying mechanisms of CIPN. Ozone treatment (O3T), which has been shown to possess antioxidant and anti-inflammatory properties, is being explored as a potential treatment for CIPN. Experimental and preliminary clinical data suggest a possible benefit in modulating oxidative stress and alleviating neuropathic symptoms [11,12]. Ozone is a potent oxidizing agent, and its therapeutic effects are thought to be mediated through controlled and transient oxidative stress. This “preconditioning” effect, also known as hormesis, can (i) activate the nuclear factor erythroid 2-related factor 2 (Nrf2) pathway, increasing cellular antioxidant responses; (ii) downregulate the Nuclear Factor kappa B (NF-κB) pathway, decreasing the production of proinflammatory cytokines and chemokines, thereby decreasing neuroinflammation; (iii) improve microcirculation and oxygen delivery to peripheral nerves, promoting tissue repair; and (iv) potentially modulate pain signaling pathways through effects on nociceptors and neurotransmitter release.

O3T has emerged as a potential therapeutic modality for a variety of conditions, including chronic ischemic or inflammatory diseases and pain. In the context of cancer, O3T is being explored as an integrative medicine approach to enhance the efficacy of radiotherapy and chemotherapy [13,14] and mitigate their side effects. While the precise mechanisms of action of ozone therapy in CIPN are still being investigated, preclinical and clinical studies have provided some evidence of its potential benefits. In this way, several preclinical and clinical studies have primarily focused on its role in reducing chemotherapy-induced side effects, such as oxidative stress, fatigue, and CIPN, as well as its potential to improve patients’ quality of life [15].

The Evidence Acquisition Terms included in the information search were as follows: survivors; chemotherapy-induced peripheral neuropathy; grade of toxicity; neuropathic pain; Nrf2; numbness and tingling; oxidative stress modulation; ozone treatment; quality of life; side effects. Bibliographic databases consulted included: MEDLINE/PubMed, SciELO, LILACS, PAHO, EMBASE, ZOTERO Ozone Health Care, WHO International Clinical Trials Registry Platform, and NIH. U.S. National Library of Medicine. The documents reviewed were published between 1995 and 2025 in English, Russian, or Spanish and included original articles, published theses, clinical reports, ongoing clinical trials, and bibliographic reviews. The exclusion criteria included a lack of free access to the complete text due to financial constraints and/or studies presenting inadequate scientific evidence.

This narrative review aims to evaluate the current preclinical and clinical evidence regarding the use of O3T in CIPN. It focuses on its potential to address key pathophysiological mechanisms, such as oxidative stress and neuroinflammation, which remain insufficiently targeted by existing treatments. This work summarizes studies published between 1995 and 2025 (including 18 experimental and 27 clinical reports) and highlights ongoing randomized controlled trials to identify research gaps and inform future clinical directions.

## 2. Chemotherapy-Induced Peripheral Neuropathy

The precise mechanisms underlying CIPN are complex and vary depending on the specific chemotherapeutic agent used [5,16]. However, several key pathways are commonly implicated, including direct damage to neuronal structures, microtubule disruption, the formation of nuclear DNA adducts, mitochondrial dysfunction, the dysregulation of ion channels, genetic susceptibility, oxidative stress, neuroinflammation, and the potential roles of the central nervous system and gut microbiota [5,7,17,18].

### 2.1. Microtubule Disruption

Microtubule-targeting agents such as taxanes (paclitaxel and docetaxel), vinca alkaloids (vincristine and vinblastine), epothilones (ixabepilone), and eribulin are well-known to induce CIPN. These drugs interfere with the normal dynamics of microtubules, which are essential components of the neuronal cytoskeleton responsible for axonal transport (they serve as tracks for the movement of organelles, vesicles, and other cellular components along the axon), the maintenance of cell shape, and nerve fiber function [5,19].

Taxanes generally stabilize microtubules, preventing the normal disassembly (depolymerization) of microtubules, which is a critical part of their dynamic behavior in cells. The stabilization of microtubules leads to their abnormal accumulation and impaired dynamics, resulting in the disruption of axonal transport, further accumulation of cellular debris, and neurofilament disorganization, ultimately leading to axonal degeneration [19,20].

Conversely, vinca alkaloids exert their effects by inhibiting the polymerization of tubulin, which diminishes microtubule mass and consequently impairs axonal transport [5,19]. This disruption is directly associated with axonal atrophy and subsequent degeneration. For instance, research involving rats has demonstrated that vincristine induces damage to the cytoskeleton of large-diameter sensory neurons and myelinated axons. Furthermore, it promotes the accumulation of neurofilaments within dorsal sensory ganglion neurons [5].

The disruption of microtubule dynamics by these agents primarily affects long and large-diameter sensory axons, contributing to the characteristic distal sensory neuropathy observed in CIPN [5,19].

### 2.2. Formation of Nuclear DNA Adducts

The formation of nuclear DNA adducts in the neurons (particularly in the dorsal root ganglion) is primarily associated with platinum-based drugs. These drugs form covalent bonds with DNA, creating adducts and nucleolar damage [10] that interfere with DNA repair, replication, and transcription (with the potential induction of apoptotic pathways) and damage several neuronal processes, such as mitochondrial dysfunction and oxidative stress regulation [9,16,21,22].

### 2.3. Mitochondrial Dysfunction

Mitochondrial damage and dysfunction are increasingly recognized as central players in the pathogenesis of CIPN induced by various chemotherapeutic agents, including platinum-based drugs, taxanes, and bortezomib. Mitochondria are essential within neurons for energy production, calcium homeostasis, and the generation of a small amount of Reactive Oxygen Species (ROS), which play essential roles in cell signaling [7,19,20].

Platinum-based drugs can directly target mitochondrial DNA and proteins, leading to impaired transcription, protein synthesis, and oxidative phosphorylation; a further decrease in ATP production and ROS overproduction; and a further increase in oxidative stress [7,19,20].

Neither vincristine nor paclitaxel appears to directly harm mitochondrial DNA. The mitochondrial dysfunction observed with vincristine is likely linked to changes in mitochondrial calcium (Ca^2+^) signaling. Conversely, paclitaxel has been shown to cause mitochondrial swelling and vacuolation within axons. Additionally, paclitaxel can promote the apoptosis of susceptible neurons via the sirtuin 1/peroxisome proliferator-activated receptor gamma coactivator 1-α (PGC-1α) pathway, which subsequently leads to an increase in oxidative stress [20,23].

### 2.4. Ion Channel Dysregulation

Chemotherapeutic agents (especially platinum compounds, taxanes, and vincristine) can directly or indirectly alter the function of various ion channels expressed in dorsal root ganglion (DRG) neurons and their peripheral axons, such as voltage-gated sodium channels (NaV), voltage-gated potassium channels (KV), voltage-gated calcium channels (CaV), and transient receptor potential (TRP) channels. This leads to neuronal hyperexcitability, spontaneous activity, and altered sensory perception, ultimately manifesting as the characteristic symptoms of CIPN [5,20].

NaV are critical for action potential generation and propagation. Oxaliplatin has been shown to induce acute cold allodynia, partly through altering the gating properties of NaV1.6 channels, leading to increased neuronal excitability and spontaneous firing [5,20]. Taxanes, such as paclitaxel, also affect NaV channels, although their effects differ from oxaliplatin. Studies have shown that paclitaxel can enhance persistent sodium currents and alter the expression of NaV subtypes, contributing to neuronal hyperexcitability [23]. Additionally, both taxanes and platins can disrupt the cytoskeleton, affecting the proper trafficking and function of NaV channels.

Beyond sodium channels, other ion channels are also involved in CIPN. KV also plays a crucial role in regulating neuronal excitability and repolarization. Oxaliplatin has been reported to inhibit KV currents in DRG neurons, further contributing to hyperexcitability [5]. In experimental models, enhanced calcium influx through CaV channels has been observed in dorsal root ganglion neurons treated with paclitaxel [17,23] and in the spinal cord after oxaliplatin [22]. It is a potential target for analgesic drugs like gabapentin and pregabalin. Finally, TRP channels, such as TRPA1 and TRPV1, are involved in sensing noxious stimuli and are sensitized in CIPN, primarily secondary to paclitaxel, contributing to cold allodynia and hyperalgesia [5,7,23].

### 2.5. Genetic Predisposition

Genetic variations can influence drug pharmacokinetics, pharmacodynamics, and individual susceptibility to CIPN [19]. For this reason, genome-wide association studies (GWAS) have identified several single-nucleotide polymorphisms (SNPs) associated with an increased risk of developing CIPN following treatment with specific chemotherapeutic agents [6,17,19].

Polymorphisms in genes encoding drug-metabolizing enzymes, drug transporters, and proteins involved in neuronal structure and function have been implicated [17,19]. For example, (i) variations in the MAPT (encoding for microtubule-associated protein tau), TUBB2A, GSK3β, CYP2C8, and CYP3A4 polymorphisms have been associated with paclitaxel-induced neuropathy [19]; (ii) NaV channels polymorphisms have been associated with oxaliplatin-induced CIPN [6]; and (iii) Actin gamma 1 (ACTG1) and Capping actin protein, Gelsolin-like (CAPG) polymorphisms have been linked to vincristine-induced neuropathy [19].

Identifying genetic risk factors could allow for personalized approaches to chemotherapy administration and CIPN prevention.

### 2.6. Oxidative Stress

Oxidative stress, characterized by an imbalance between the production of ROS and antioxidant defense mechanisms, plays a significant role in the development and maintenance of CIPN. Chemotherapeutic agents, such as platinum drugs, taxanes, and vinca alkaloids, have all been shown to induce the generation of ROS and further oxidative stress in peripheral neurons and glial cells, leading to damage to lipids, enzymes, proteins, and DNA [5,17,24].

Oxidative stress induces (i) mitochondrial DNA damage with further energy deficit and calcium dysregulation in neurons; (ii) protein nitration and lipid peroxidation, which result in the disruption of microtubules and cytoskeleton, the disruption of axonal transport, axonal degeneration, and demyelination of peripheral nerve; (iii) activation of proinflammatory pathways and neuroinflammation; and (iv) apoptosis of sensory neurons [5,17,20,24,25].

Additionally, oxidative stress can activate TRPA1 channels (which are redox-sensitive), contributing to inflammation and pain signaling. TRPA1, in collaboration with other TRP channels, is also involved in mechanical and cold hypersensitivity induced by cisplatin and oxaliplatin [5,17].

### 2.7. Neuroinflammation

Neuroinflammation results from nociceptor sensitization due to damage to mitochondrial DNA transcription, the release of ROS, and demyelination of peripheral neurons [9,24]. Neuroinflammation plays a critical role in CIPN pathogenesis, characterized by the activation of glial cells (Schwann cells, macrophages, astrocytes, and microglia) and the subsequent release of proinflammatory cytokines, interleukins (IL), and chemokines [18,26]. Spinal astrocytes, rather than microglia, have been described as contributors to painful paclitaxel-induced neuropathy [26].

Chemotherapeutic agents can directly activate these glial cells, triggering the release of inflammatory mediators such as tumor necrosis factor-alpha (TNF-α), IL-1β, IL-6, and IL-8, as well as the decreased production of anti-inflammatory IL [5,17,20,23,25,27]. Additionally, proinflammatory cytokines can exert adverse, multifaceted effects in CIPN through the progression of cancer, the activation and maintenance of mast cells (along with the release of serotonin and histamine), and the dysfunction of natural killer (NK) cells [18]. These inflammatory mediators can sensitize nociceptors in the peripheral nervous system, contributing to the development of pain, hyperalgesia, and altered sensitivity [17,27].

Chemokine signaling (such as CX3CL1-mediated macrophage activation) and chemokine ligands (such as CCL2 and its receptor CCR2) can contribute to dorsal root ganglion neuronal apoptosis and CIPN symptoms induced by paclitaxel and oxaliplatin [9,19,22,26]. Galectin-3, derived from Schwann cells, plays a key role in maintaining peripheral nerve integrity, and it is implicated in myelination, nerve regeneration, and the inflammatory response following injury. This inflammatory action has also been involved in pro-nociceptive roles in taxane-induced neuropathy [28]. Another experimental study described that CIPN induced by oxaliplatin was associated with the overexpression of endogenous coagulation tissue factor (TF) and matrix metalloproteinase (MMP)-9/2 (Gelatinase B) activity in sciatic nerve and blood. This work also described the increased release of Heat Shock Protein 70 (HSP70) from macrophages, activation of the Toll-like receptor 4 (TLR4)/phosphorylated p38 Mitogen-Activated Protein Kinase (MAPK p38) pathway, and an increase in hypoxia-inducible factor-1α (HIF-1α), supporting the role of these immune-related pathways and microcirculation disturbances in the neuroinflammation process leading to CIPN [29].

In this context, the infiltration of macrophages into the dorsal root ganglion induced by paclitaxel [26] is associated with CIPN. Furthermore, the increase in the levels of circulating helper (CD4+) T lymphocytes and cytotoxic (CD8+) T lymphocytes, which can further activate other immune cells (e.g., B cells or macrophages), is induced by oxaliplatin [20]. In paclitaxel-treated wild-type mice, increased proliferation of helper T-lymphocytes has also been described, but with a decrease in cytotoxic T-lymphocytes [23].

Thus, altered immune system regulation seems to result in neuroinflammation processes, and the interplay between neuronal damage and neuroinflammation creates a vicious cycle that perpetuates CIPN symptoms [18,27].

### 2.8. The Role of the Brain in CIPN

While CIPN is defined as damage to the peripheral nervous system, emerging evidence suggests that the central nervous system also plays a role in the perception and maintenance of CIPN-related pain. Central sensitization, characterized by increased excitability of neurons in the spinal cord and brain, can amplify pain signals and contribute to chronic pain in CIPN [18,30]. This supports the poor correlation between nerve conduction tests and CIPN symptoms reported by patients [30,31].

Omran et al. [30] reviewed 29 articles related to this topic: 3 in humans, 2 in macaque monkeys, and 24 in rodents. Overall, their review suggests that CIPN positively correlates with the following four factors.

(i) Brain hyperactivity (particularly the insula). The hypothesis is that brain amplification of peripheral inputs could explain CIPN symptoms, including hyperalgesia, increased sensitivity to pain, and allodynia (the experience of pain with normally non-painful stimuli). Hyperexcitability in the neurons of the spinal cord and the dorsal root ganglion can also be produced by neurotoxic chemotherapy. In human studies, evaluation using functional and perfusion magnetic resonance imaging (MRI) showed that (a) CIPN was associated with brain hyperactivity in response to painful stimuli in sensory regions (S2/insula), whereas a reduction in CIPN-symptoms is associated with a reduction in brain activity in the insula, and (b) the anterior default mode network showed greater resting perfusion but reduced activity after painful stimuli.

(ii) A reduction in gamma-aminobutyric acid (GABA) levels in the brain. This leads to decreased inhibitory signaling, whereas experimental activation of the GABAergic system reduces and/or reverses CIPN symptoms. Alterations in GABAergic signaling in the spinal cord may also contribute to CIPN. However, clinically, GABA analogs (such as gabapentin) are used in neuropathic pain but have common side effects and have not demonstrated clear benefits in CIPN.

(iii) Neuroinflammation. CIPN is frequently associated with increased levels of proinflammatory cytokines (such as IL-1β, IL-6, and TNFα) and their receptors in the brain and spinal cord. In experimental models, reducing proinflammatory cytokines, blocking proinflammatory cytokine receptors, or restoring anti-inflammatory cytokines (such as IL-4, IL-10, and IL-13) can decrease CIPN symptoms. In this process, mast cells and NK cells play a relevant role [18].

(iv) Overactivation of G-coupled protein receptors (GPCRs) and MAPK pathways in the brain. These factors appear to be involved in CIPN. In experimental models of oxaliplatin CIPN, the effects of morphine and oxycodone were blocked using GPCRs/MAPK inhibitors.

### 2.9. The Role of the Gut Microbiota

The gut microbiota is a complex ecosystem residing within the gastrointestinal tract. It engages in bidirectional communication with the host’s nervous and immune systems through the gut–brain axis, which involves a variety of signaling molecules and pathways, allowing the gut microbiome to influence systemic inflammation and immune responses, which can, in turn, affect the peripheral nervous system. Chemotherapeutic drugs, while targeting cancer cells, can also disrupt the delicate balance of the gut microbiota, leading to dysbiosis and potentially increasing intestinal permeability. This disruption can result in an imbalance in the production of bacterial metabolites, which may then contribute to the activation of immune and glial cells and the subsequent development of neuroinflammation, a crucial element in the pathogenesis of CIPN [32,33].

Experimental models have demonstrated a causal link between changes in the gut microbiota composition and sensitivity to oxaliplatin- and paclitaxel-induced CIPN. A study described how mice pretreated with a cocktail of antibiotics to deplete their gut bacteria showed a decrease in oxaliplatin-induced hyperalgesia. Conversely, when the microbiota of germ-free mice was restored through fecal transplantation from specific pathogen-free mice, the protective effect against mechanical hyperalgesia was reversed. Furthermore, the study’s findings suggest that these effects are partially mediated by lipopolysaccharides from gut microbiota, which are ligands of TLR4, a key receptor in the immune system that recognizes bacterial components [34]. On the other hand, a study with paclitaxel-treated mice showed a reduction in specific bacterial species, such as the abundance of *Akkermansia muciniphila* (which is involved in maintaining the integrity of the gut barrier), leading to increased systemic exposure to bacterial metabolites and products that might contribute to neuroinflammation and neuropathic pain. Furthermore, the transfer of gut microbiota from pain-sensitive mice to pain-resistant mice, and vice versa, influenced the susceptibility of the recipient mice to paclitaxel-induced pain. This, along with the specific antibiotic depletion studies conducted in this study, also supports the significant role of gut microbes in CIPN [35].

A pilot study investigated the efficacy of minocycline, an antibiotic with potential anti-inflammatory effects, in preventing CIPN in patients receiving paclitaxel. While the study did not find evidence to support the use of minocycline for CIPN prevention, it suggested a potential benefit in reducing acute pain associated with paclitaxel treatment [36]. In this study, minocycline (200 mg on day 1, then 100 mg twice daily) was compared to a placebo, and there were no significant differences between groups in the overall sensory neuropathy scores or individual symptoms (such as tingling, numbness, or burning pain) measured using the EORTC QLQ-CIPN20 instrument. The lack of effect may be attributed to the complex and multifactorial pathophysiology of CIPN in humans, which involves mechanisms beyond neuroinflammation, such as mitochondrial dysfunction and alterations in ion channels, potentially limiting the impact of anti-inflammatory agents like minocycline. Notably, while minocycline did not prevent chronic neuropathy, it was associated with a reduction in acute pain syndrome and fatigue during paclitaxel treatment, suggesting a possible symptomatic benefit in these domains. However, these findings do not support the use of minocycline for preventing CIPN, and further research is needed to clarify its potential role, if any, in managing acute chemotherapy-related symptoms. This aligns with the broader clinical literature, which indicates that no pharmacologic agent, including minocycline, is currently recommended for CIPN prevention, and only duloxetine is supported for established painful CIPN [37].

Current research indicates that the gut microbiome plays a role in the onset and advancement of CIPN, suggesting that microbiota-targeted therapies (e.g., prebiotics, probiotics, or fecal microbiota transplantation) may be beneficial in managing CIPN [33]. However, further research is required to fully elucidate the intricate mechanisms involved and to translate these findings into effective clinical treatments.

## 3. Summary of Clinical Manifestations and Current Management Strategies of CIPN

### 3.1. Clinical Manifestations

The clinical manifestations of CIPN primarily involve sensory disturbances, which can include pain, tingling, numbness, and a “glove and stocking” distribution of symptoms. Motor and autonomic changes may also occur, though they are less common. The combination of oxidative stress and neuroinflammation leads to increased neuronal excitability and altered ion channel expression in dorsal root ganglion neurons, further contributing to the pain and sensory disturbances experienced by patients [38,39]. Understanding these mechanisms is essential for developing effective preventive and therapeutic strategies for CIPN.

### 3.2. Current Management Strategies

Current management strategies for CIPN focus on symptom relief and include pharmacological and non-pharmacological approaches. However, these strategies have limited effects, particularly considering the roles of oxidative stress and neuroinflammation in the pathogenesis of CIPN.

Among the pharmacological treatments, the most relevant is duloxetine, the only evidence-based treatment [1]. However, its effectiveness is limited to CIPN-related pain (no to numbness and tingling), and “benefits equal harms” with a “Strength of recommendation: moderate” because of the modest magnitude of its benefit, according to ASCO [1]. A multicenter randomized controlled trial (RCT) on a 10-point scale showed a 1.06-point pain reduction compared to a 0.34-point reduction in the placebo group, and the effect was lost one week after stopping duloxetine [40]. Other drugs have shown promising results, but they are not recommended outside clinical trials: (i) Gabapentinoids (gabapentin and pregabalin) are commonly used for neuropathic pain, but their efficacy in CIPN is inconsistent and often inadequate, (ii) tricyclic antidepressants (amitriptyline and nortriptyline) are used off-label for CIPN, but evidence supporting their use is weak, and they have significant side effects; (iii) topical agents (capsaicin and lidocaine) may provide localized pain relief, but their overall benefit in CIPN is limited [1,12,41].

Non-pharmacological treatments have shown promising results in established CIPN in some studies, with improvement in symptoms or quality of life, such as exercise, acupuncture, and scrambler therapy. For emerging therapies such as scrambler therapy and exercise, the evidence base is evolving but remains insufficient for formal recommendations outside of clinical trials [42,43]. Scrambler therapy and exercise target neural signaling and neuroregeneration, respectively, while ozone therapy targets oxidative and inflammatory pathways, as well as microcirculatory effects. In addition, other novel approaches (such as acupuncture, neurofeedback, and various pharmacological agents) have not demonstrated consistent benefits in high-quality trials and are not recommended by ASCO [1]. Therefore, further RCTs are required [1,12]. Similarly, no evidence-based recommendations can be made for the prevention of CIPN, including antioxidants, the above-mentioned drugs (gabapentinoids and tricyclic antidepressants), or non-pharmacological treatments (such as cryotherapy, acupuncture, or exercise) [1].

The limitations of the current management strategies for CIPN include the following factors: (i) A lack of FDA-approved drugs, no drugs are specifically approved for CIPN, and current treatments often provide only partial relief [44]. (ii) Mechanistic understanding, the precise mechanisms of CIPN, including the roles of oxidative stress and neuroinflammation, are not fully understood; they can be different for different chemotherapy drugs, and with different responses to CIPN treatment, hindering the development of targeted therapies [45]. (iii) The heterogeneity of CIPN. Variability in symptoms and responses to treatment among patients complicates management and necessitates personalized approaches [39].

## 4. Ozone Treatment: Background and Mechanisms of Action

Ozone (O_3_) is more reactive and soluble in water and plasma than oxygen (O_2_). O3T consists of administering an O_3_/O_2_ gas mixture obtained from medical-grade oxygen using a medically certified ozone-generating device as integrative medicine. O3T should follow a precise medical protocol and should be prescribed by a medical doctor. Because of its short half-life (40 min at 20 °C and 25 min at 30 °C), ozone cannot be encapsulated or stored and must be generated and used in situ. The usual O_3_/O_2_ concentration (µg of O_3_ per mL of O_2_) for clinical use ranges from 10 to 60 µg/mL in an O_3_/O_2_ gas mixture, where most is O_2_, and only a small percentage (0.5–0.05%) corresponds to O_3_ [11,46].

O3T can be administered through various routes, each with specific applications and dosage considerations focused on local or systemic effects. They are focused on local effects: (i) Local injections, where O_3_/O_2_ is injected directly into joints, paravertebrally, or intradiscally for musculoskeletal conditions and pain management [47,48]. (ii) Topical applications, where ozone gas, ozonated oil, or ozonated water are applied directly to the skin (for dermatological conditions) or different mucous membranes (vaginal, vesical, oral) for superficial infections and wound healing [48]. This included alterations secondary to chemotherapy, such as mucositis [49,50,51] or skin necrosis after extravasation, as well as radiotherapy-induced toxicity, including dermatitis [14], oral [52], and vesical [53] mucositis. When focusing on inducing a systemic effect, the two most relevant routes are (i) major autohemotherapy (MAHT), which involves drawing 100–200 mL of the patient’s blood, ozonating it ex vivo with a precise O_3_/O_2_ concentration, and then reinfusing it back into the patient in a 30–60 min procedure; and (ii) rectal insufflation, where 150–300 mL of O_3_/O_2_ gas mixture is slowly insufflated into the rectum in a 20–30 min procedure [46,48,54,55].

There is evidence of oxidative stress in patients with CIPN. Multiple chemotherapeutic agents, including platinum compounds, taxanes, vinca alkaloids, and bortezomib, induce chronic oxidative stress and mitochondrial dysfunction and increase the production of ROS, which contribute to neuronal injury, apoptosis, and demyelination in CIPN. This pathophysiological mechanism is well established and underlies the rationale for exploring antioxidant-based therapies in CIPN management [25]. High doses of ozone therapy can be harmful. While controlled, low-dose ozone therapy may induce adaptive antioxidant and anti-inflammatory responses, excessive ozone exposure acts as a potent oxidant, increasing oxidative stress, lipid peroxidation, and cellular damage, which can potentially exacerbate neurotoxicity and tissue injury. Therefore, the safety and efficacy of O3T in CIPN are highly dose-dependent, and inappropriate dosing may worsen oxidative damage rather than provide benefit.

The central concept underlying the therapeutic use of ozone is the principle of hormesis, which posits that low doses of a stressor can elicit beneficial biological responses, often opposing the effects of high doses. In the context of O3T, a small, precisely controlled ozone dose applied to the blood or rectal mucous can induce transient and mild oxidative stress, which is counteracted by the body’s antioxidant defense mechanisms. This acute, transitory oxidative stress is crucial for triggering biological effects without causing toxicity. Ozone concentrations that are too low may be ineffective because endogenous antioxidants can directly eliminate all the produced ROS, potentially eliciting only a placebo effect. In contrast, excessively high concentrations can exceed the organism’s adaptation capacity, potentially leading to adverse side effects. Therefore, achieving a therapeutic result depends on using precisely controlled ozone concentrations just above the threshold level required to elicit an adaptive biological response [11,12,46,48,54,55,56].

When gaseous ozone comes into contact with biological fluids such as blood or rectal mucous, it rapidly reacts with organic compounds, forming ROS and lipid oxidation products (LOPs) that interact with various cellular and molecular targets. These include hydrogen peroxide (H_2_O_2_) and aldehydes like 4-hydroxynonenal (4-HNE), which act as second messengers, mediating further adaptive responses within the body at the cellular (the former) and systemic (the latter) levels. There are three key mechanisms.

### 4.1. Activation of the Antioxidant System

Appropriate O3T induces a controlled, mild, and transient oxidative stress that produces an adaptive response. This is mediated by the upregulation of transcription factors such as Nrf2, which increases the production of antioxidant enzymes (such as superoxide dismutase (SOD), catalase (CAT), and glutathione peroxidase (GPx); glutathione S-transferases (GSTs); and heme oxygenase-1 (HO-1)) and a further reduction in oxidative stress [57,58,59,60,61].

### 4.2. System Modulation of Inflammation

As described in Section 2.6 and Section 2.7, oxidative stress and neuroinflammation are relevant mechanisms involved in the pathogenesis of CIPN, where the imbalance between the antioxidant pathway regulated by Nrf2 and the proinflammatory pathway mediated by NF-κB plays a central role. Chemotherapeutic agents can suppress Nrf2 activity and activate NF-κB, leading to increased oxidative stress and inflammation in peripheral nerves [24,25,62].

O3T has been shown to modulate the NF-κB pathway, which is involved in the inflammatory response. The dual role of redox activation of Nrf2 and NF-κB shows that while Nrf2 activation confers cell protection, NF-κB is involved in deleterious effects within the cell [63]. Additionally, it has been demonstrated that Nrf2 overexpression can inhibit NF-κB activation, suggesting an antagonistic relationship between these pathways [64].

Subsequently, O3T exhibits a dual role in inflammation, depending on the dosage. (i) Low to moderate ozone concentrations have been shown to have anti-inflammatory effects, potentially by interfering with proinflammatory signaling pathways such as NF-κB. Additionally, H_2_O_2_ can enter mononuclear cells and modulate the NF-κB pathway. In this way, O3T can potentially modulate the production of proinflammatory cytokines (IL-1β, IL-6, IL-8, TNFα, and IFN-γ) and influence immune cell activity. (ii) While high ozone doses might activate NF-κB and promote inflammation, therapeutic doses of ozone can block the NF-κB signal, thus reducing inflammation [11,46,48,54,55,56].

Other potential mechanisms of action of O3T include (i) downregulation of iNOS [65], which could mitigate macrophage-induced overexpression after bortezomib treatment [66]; (ii) an increase in transforming growth factor beta 1 (TGF-β1) [67], which could enhance the effect of endogenous opioids [68]; and (iii) upregulation of the adenosine 5′-monophosphate-activated protein kinase (AMPK)/suppressors of cytokine signaling 3 (SOCS3), which can inhibit the TLR4-mediated inflammatory pathway [69].

### 4.3. Improvement in Microcirculation and Oxygen Delivery

Several studies have shown that O3T can release nitric oxide in the vascular endothelium [70] and improve rheological properties [71,72], tissue oxygenation [72], blood flow [73], and metabolism [73].

By reducing inflammation and oxidative stress, ozone therapy helps restore normal vascular function, thereby enhancing oxygen delivery to peripheral nerves and promoting tissue repair [11,12]. Additionally, as already mentioned, O3T activates the AMPK pathway, improving endothelial nitric oxide synthase (eNOS) activity. Enhanced eNOS activity increases nitric oxide production, a potent vasodilator that improves blood flow and oxygen delivery to peripheral tissues [74,75]. Moreover, AMPK activation leads to the phosphorylation and activation of bisphosphoglycerate (BPG) mutase, the enzyme responsible for producing 2,3-BPG. This pathway is beneficial in hypoxic conditions by increasing 2,3-BPG levels and enhancing oxygen release from erythrocytes [76]. 2,3-BPG is a crucial regulator of hemoglobin’s oxygen-binding affinity. By increasing the levels of 2,3-BPG, ozone therapy decreases the affinity of hemoglobin for oxygen, facilitating the release of oxygen from hemoglobin to the tissues. This enhanced oxygen release improves tissue oxygenation and helps counteract hypoxia.

Hypoxia is a component of CIPN; chemotherapeutic agents such as platinum compounds, taxanes, and bortezomib can induce mitochondrial dysfunction and oxidative stress, leading to hypoxia in peripheral nerves. This hypoxia results from impaired mitochondrial respiration and reduced ATP production, compromising the energy supply necessary for neuronal function and survival [6,7]. Additionally, the disruption of microtubule stability and axonal transport dynamics caused by these agents further exacerbates hypoxic conditions by impairing the delivery of essential nutrients and oxygen to peripheral nerves [38]. The resultant hypoxia contributes to neuronal damage, axonal degeneration, and clinical manifestations of CIPN, such as pain, numbness, and tingling [27,77].

**Figure 1:**
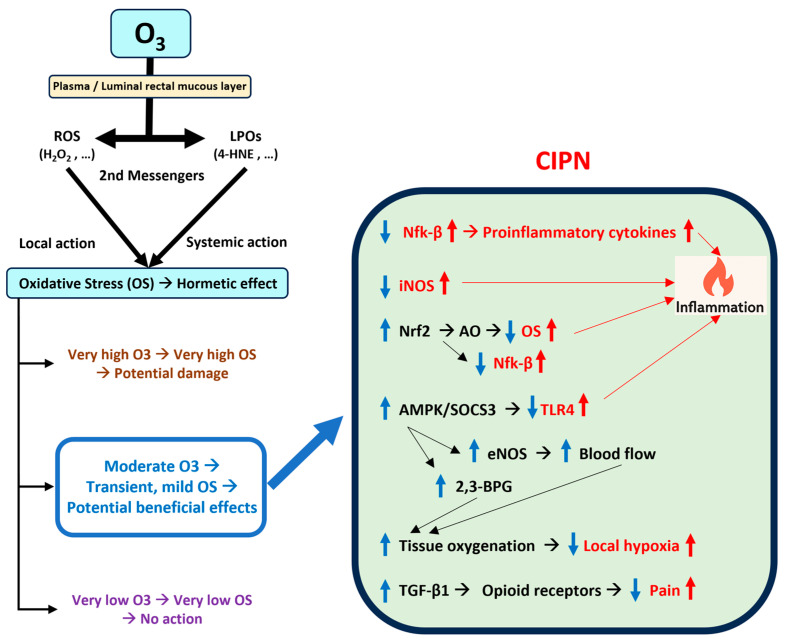
Summary of the potential effects of ozone therapy on chemotherapy-induced peripheral neuropathy. Abbreviations: 4-HNE: 4-hydroxynonenal; AMPK: Adenosine -monophosphate-activated protein kinase; AO: Antioxidants; CIPN: Chemotherapy-induced peripheral neuropathy; eNOS: Endothelial nitric oxide synthase; H_2_O_2_: Hydrogen peroxide; iNOS: Inducible nitric oxide synthase; LPOs: Lipoperoxides; Nfk-β: Nuclear factor kappa B; Nrf2: Nuclear factor erythroid 2-related factor 2; O_3_: Ozone; OS: Oxidative stress; ROS: Reactive oxygen species; SOCS3: Suppressors of cytokine signaling 3; TGF-β1: Transforming growth factor beta 1; TLR4: Toll-like receptor 4. Upward arrows: increase; downward arrows: decrease; in red: effects of CIPN; in blue: effects of therapeutic ozone.

Figure 1 illustrates a schematic representation of the mechanisms of action of ozone.

## 5. Ozone Treatment: Preclinical and Clinical Studies

### 5.1. Preclinical Studies

Two reviews have described previous preclinical research evaluating the role of O3T to prevent or treat several chemotherapy-induced side effects (including cisplatin-induced ototoxicity [78]), drug-induced diabetic neuropathy, and injury after sciatic nerve cutting. The reviewed studies showed a beneficial effect of O3T, and most showed that the modulation of oxidative stress mediated the effect. However, those studies did not directly address CIPN [12,56].

Fortunately, a recent and relevant study by Zhang et al. [79] provided additional insights into the potential mechanisms by which ozone therapy may alleviate CIPN. Using an oxaliplatin mouse model to evaluate the effects of O3T on CIPN, this study demonstrated that (i) O3T prevented the oxaliplatin-induced increase in MMP-9 and prevented oxaliplatin-induced mechanical allodynia, which is associated with decreased plantar blood flow (measured via laser speckle contrast imaging); (ii) O3T decreased the oxaliplatin-induced overexpression of TF in blood and the sciatic nerve, limiting upstream TLR4/p38 activation by TF; (iii) O3T upregulated AMPK-SOCS3 axis and inhibited TF and p38 expression, suggesting that O3T could inhibit the TLR4/p38/TF signaling pathway by activating the AMPK-SOCS3 axis; (iv) O3T inhibited microglia activation and decreased FBJ murine osteosarcoma viral oncogene homolog (c-FOS) and calcitonin gene-related peptide (CGRP) expression in the spinal dorsal horn; and (v) finally, using electron microscope analysis, this study demonstrated that O3T prevented oxaliplatin-induced sciatic nerve demyelination [79].

### 5.2. Clinical Studies

Our group has published several reports about the potential benefits of O3T in the management of side effects of cancer treatment in patients with delayed wound healing after surgery or radiotherapy [14], radiation-induced brain damage [73], hematuria [53], rectal bleeding [80], and chronic pelvic pain [81], with some results evaluated at 9 months [81] and 100 months [80] of follow-up after the end of O3T, which suggest that some effects of O3T may be maintained for a long time.

Despite the results of several animal models, clinical evidence supporting the use of ozone therapy for CIPN is limited. In this review, we summarize the main findings from our recent reports investigating the potential of O3T in managing various aspects of CIPN. In the following four studies, O3T was administered via rectal insufflation in 40 planned sessions over 4 months, with progressive increases in O_3_/O_2_ concentration (from 10 to 30 μg/mL) and gas volume (from 180 to 300 mL). These studies explored the effects of O3T on (i) chronic pain, (ii) numbness and tingling and the related grade of toxicity, (iii) anxiety and depression, and (iv) overall health-related quality of life (HRQOL) in patients experiencing CIPN.

**Figure 2:**
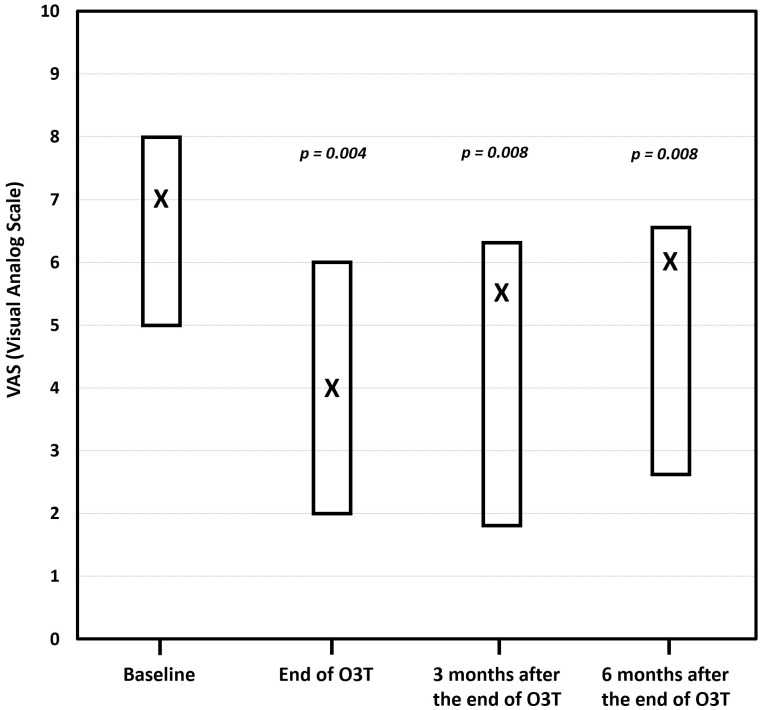
The effect of ozone treatment (O3T) on chronic pain according to the visual analog scale (VAS). Compared to the baseline value, the median VAS pain score was significantly lower at the end of O3T (*p* = 0.004), 3 months (*p* = 0.008), and 6 months (*p* = 0.008) after the end of O3T. Box: Quartiles 1 and 3; X: median value (adapted from [82]).

**Figure 3:**
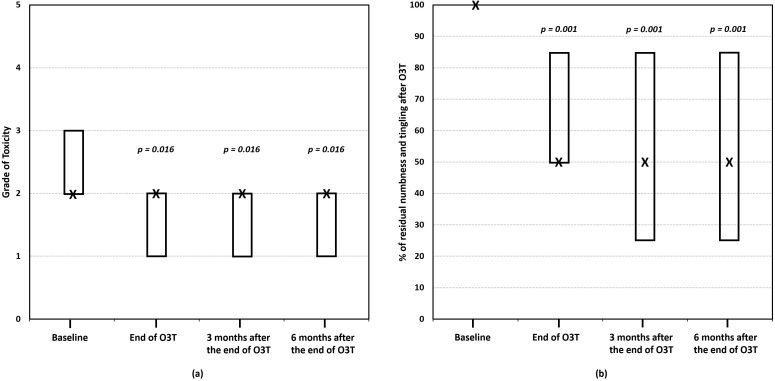
The effect of ozone treatment (O3T) on numbness and tingling. (**a**) The grade of toxicity (for paresthesia) according to the Common Terminology Criteria for Adverse Events (CTCAE v.5.0) scale of the National Cancer Institute of EEUU, ranging from 0 (no toxicity) to 5 (death). (**b**) The percentage of residual numbness and tingling (baseline value = 100%). There was a significant improvement in both parameters after O3T, which was maintained over 6 months. Box: Quartiles 1 and 3; X: median value (adapted from [83]).

**Figure 4:**
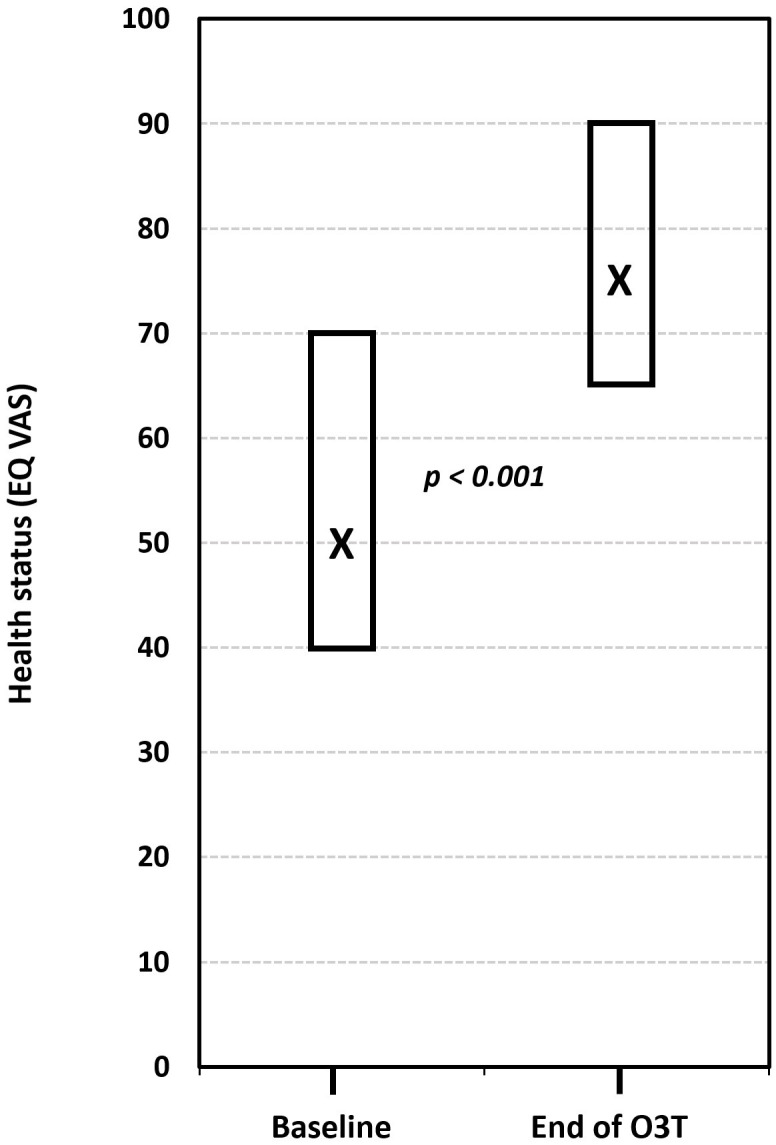
The effect of ozone treatment (O3T) on self-perceived general health status (EQ-VAS) in the EQ-5D-5L™ questionnaire (scored from “0” (worst health status) to “100” (best health status)). There was a significant improvement in the self-perceived EQ-VAS after O3T (*p* < 0.001). Box: Quartiles 1 and 3; X: median value (adapted from [84]).

**Figure 5:**
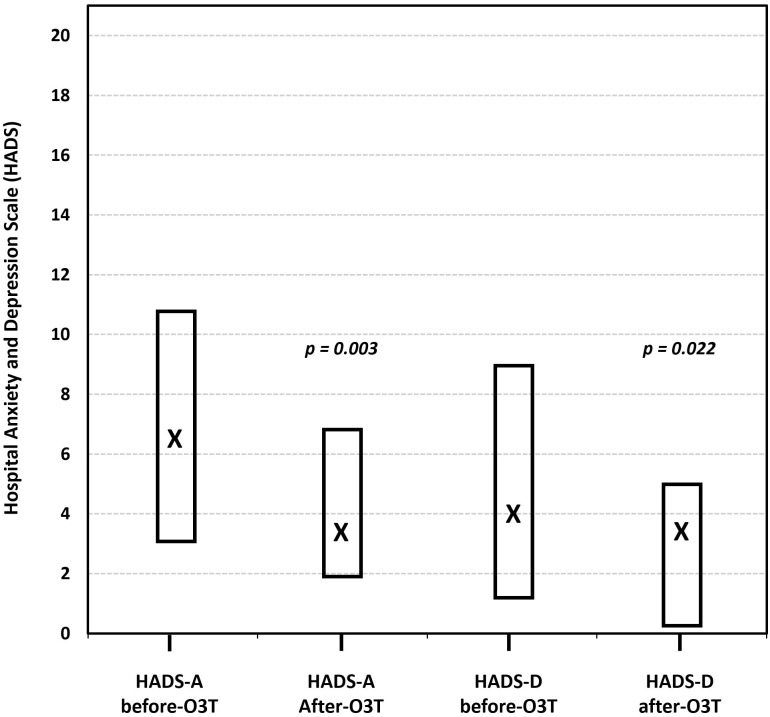
The effect of ozone treatment (O3T) on anxiety and depression, according to the Hospital Anxiety and Depression Scale (HADS), ranging from 0 (best) to 21 (worst). Both symptoms were significantly lower after O3T. Box: Quartiles 1 and 3; X: median value (adapted from [85]).

(i) Effect on chronic pain. The first report described the long-term improvement in painful CIPN after O3T. This study included 11 different locations (hands and/or feet) in seven patients suffering from chronic and painful CIPN with grade II or grade III toxicity according to the Common Terminology Criteria for Adverse Events (CTCAE) v.5.0 scale of the National Cancer Institute of the EEUU. The median duration of symptoms before O3T was 12 months. Pain was assessed using the visual analog scale (VAS) before, at the end of, and 3 and 6 months after the completion of O3T. The results indicated clinically relevant pain improvement in six out of the seven patients. The median VAS score significantly decreased from 7 (range: 5–8) before O3T to 4 (range: 2–6) at the end of O3T (*p* = 0.004). This improvement was sustained at 3 months (median VAS: 5.5, range: 1.8–6.3, *p* = 0.008) and 6 months (median VAS: 6, range: 2.6–6.6, *p* = 0.008) after the end of O3T. Furthermore, the grade of toxicity according to the CTCAE improved in half of the patients, although it was not statistically significant (*p* = 0.125) (Figure 2) [82].

(ii) Effect on numbness and tingling. Our most recent study was specifically focused on the long-term effects of O3T in 15 patients with persistent numbness and tingling secondary to CIPN (grade 2 or grade 3 according to the CTCAE). The median duration of symptoms before O3T was 21 months. O3T was planned as the first study. The grade of CIPN-toxicity and the self-reported decrease in numbness and tingling were assessed before, at the end of, and 3 and 6 months after the end of O3T. This study found that, after O3T, 47% of patients experienced a decrease in the grade of CIPN toxicity (*p* = 0.016), and 67% of patients reported a decrease in the basal self-perception of numbness and tingling ≥50% (*p* = 0.002). These improvements (and statistical significance) were maintained at 3 and 6 months after the end of O3T. The median percentage of residual numbness and tingling significantly decreased and remained stable over the follow-up period (*p* < 0.001). There was a significant correlation between a lower grade of toxicity (grade 2 versus grade 3) and a lower percentage of residual numbness and tingling at the end of O3T and after 3 and 6 months (rho ≥ 0.651, *p* ≤ 0.011) (Figure 3) [83].

(iii) Effect on HRQOL and the grade of toxicity. This study described the effect of O3T on HRQOL and the grade of toxicity in 26 cancer survivors with chronic side effects of radiotherapy and chemotherapy, including 15 patients with CIPN. HRQOL was assessed using the five dimensions of the Spanish version (v1.0, 2009) of the EQ-5D-5L™ questionnaire (mobility, self-care, activities of daily living, pain or discomfort, and anxiety or depression). The five-dimension score ranges from 1 (“I have no problems”) to 5 (“I have a lot of problems”). The EQ-5D-5L™ also includes a VAS measuring self-perceived general health status (EQ-VAS), which is scored from “0” (worst health status) to “100” (best health status). Patients treated because of CIPN showed a significant improvement in the EQ-5D-5L index (*p* < 0.001) and in all its dimensions after O3T (*p* < 0.05 for each dimension). The self-evaluation of health status using the VAS also significantly improved from a median value of 50 (interquartile range: 40–70) before O3T to 75 (interquartile range: 65–90) at the end of O3T (*p* < 0.001) (Figure 4) [84].

(iv) Effect on anxiety and depression. Another study investigated the effects of O3T on anxiety and depression in 16 patients treated because of refractory symptoms of severe diseases, including a subset of 13 patients with side effects of cancer treatment, 8 of whom had CIPN. Depending on the patient’s symptoms, O3T was administered either systemically (via rectal insufflation or MAHT) and/or locally. Anxiety and depression were assessed using the Hospital Anxiety and Depression Scale (HADS) before and after O3T. The authors also utilized the anxiety/depression dimension and the self-perceived general health status (EQ VAS) of the Spanish version (v1.0, 2009) of the EQ-5D-5L™ questionnaire. The study found a significant decrease in both anxiety and depression severity levels after O3T. The median HADS-Anxiety score decreased from 6.5 to 3.5 (*p* = 0.003), and the median HADS-Depression score decreased from 4 to 3.5 (*p* = 0.022) at the end of O3T. Similarly, the anxiety/depression dimension of the EQ-5D-5L™ showed a significant improvement (*p* = 0.047). HADS-Anxiety and HAD-Depression were significantly correlated with the anxiety/depression dimension of the EQ-5D-5L (*p* < 0.004) and inversely correlated with the self-perceived general health status (Figure 5) [85].

A summary of the most relevant clinical studies and reviews on ozone therapy for CIPN is presented in Table 1.

The findings from these studies show two relevant characteristics of O3T when compared with other potential therapies. (i) The magnitude of clinical improvement. The median pain reduction was 3 points for O3T compared with the 1-point reduction (on a 10-point scale) described for duloxetine [40]; meanwhile, a decrease of 50% (or greater) in numbness and tingling was achieved in two-thirds of patients. (ii) The duration of the clinical effect of O3T. The sustained effect for at least 6 months in these studies compares favorably with other CIPN interventions, such as duloxetine for painful CIPN (where half the modest effect was lost within one week post-treatment) [40], or improvements observed after scrambler therapy (8 weeks) [86] and after acupuncture (12 weeks) [87]. The sustained magnitude and duration of O3T effects align with previously documented long-term benefits in radiation-induced toxicities, such as pelvic pain (9 months) [81] and refractory hemorrhagic proctitis (several years) [80].

However, these preliminary reports have significant limitations. (i) The first two studies (focused on pain [82] and numbness and tingling [83]) only evaluated patients with CIPN. The third study (focused on QOL and the grade of toxicity [84]) evaluated cancer patients with side effects of radiotherapy and chemotherapy, although in this review, we only described data from that work about patients with CIPN. (ii) However, the last paper (focused on anxiety and depression [85]) shows the results of O3T in a mixed group of 16 patients, 8 with CIPN and 8 with other refractory symptoms that could potentially confound the specific effects on CIPN patients. (iii) Additionally, the evaluation of the effects of O3T on anxiety and depression requires further and specific analysis because this effect could be (at least partially) related to the improvement in physical symptoms, such as pain, numbness, and tingling in patients with CIPN. (iv) The self-reported percentage of improvement in numbness and tingling is a non-validated outcome for CIPN. However, it is frequently used for evaluation in clinical practice. It is similar to the VAS, which introduces additional patient-reported outcomes (PROs) focused on the patient’s experience with the symptom in their everyday life. (v) The main limitations are the small patient sample sizes and the observational nature of these studies, which lack a control group. (vi) Finally, the two most relevant limitations are that these studies included a small number of patients and were observational studies without a control group. Although a placebo effect cannot be completely ruled out, the long duration of symptoms before O3T (a median of 21 months for the numbness and tingling study) and the sustained improvements observed in some studies (up to 6 months after treatment completion) reduce the likelihood of this being the case. This means that a placebo effect cannot be entirely ruled out, although the long duration of symptoms before O3T (a median of 21 months for the numbness and tingling study) and the sustained improvements observed in some studies (6 months after treatment) make this less likely.

Despite the promising preliminary findings, the current clinical evidence supporting the use of ozone therapy for CIPN remains limited. Most available studies involve small sample sizes, lack control groups, and are subject to potential placebo effects. Additionally, there is considerable heterogeneity in study design, the heterogeneity of O3T protocols (dose and route), and outcome measures (e.g., a lack of biomarkers to predict response), which makes it difficult to draw firm conclusions or compare results across studies. These limitations underscore the urgent need for well-designed, large-scale randomized controlled trials to validate the efficacy and safety of ozone therapy in this context.

On the other hand, we must consider that the management of CIPN remains challenging, with limited effective therapeutic options and limited evidence. As mentioned in Section 3.2, duloxetine is currently the only well-established treatment for painful CIPN recommended by ASCO [1]. For symptoms such as numbness and tingling, there is no evidence-based treatment. While duloxetine has shown some potential in exploratory analyses to decrease non-painful CIPN symptoms, the evidence is not robust enough to establish it as a standard treatment. Other pharmacological options, such as tricyclic antidepressants, gabapentinoids, and topical agents, have been investigated. However, their efficacy remains inconclusive, and they are not widely recommended due to limited benefits and potential side effects [1,41].

## 6. Conclusions

CIPN remains a significant clinical challenge, profoundly affecting cancer survivors’ quality of life. Furthermore, there are limited effective therapeutic options, especially for non-painful symptoms such as numbness and tingling. Therapies that target the underlying pathophysiology of CIPN rather than solely managing symptoms are required. O3T has the potential to address several mechanisms involved in the pathophysiology of CIPN.

This review summarizes the current understanding of CIPN pathogenesis, the limited treatment options, the proposed multifaceted mechanisms of O3T, and the preliminary results of our clinical studies.

Despite their limitations, such as small sample sizes and a lack of control groups, our preliminary results suggest that systemic O3T may offer tangible benefits for patients with established chronic CIPN in terms of pain, severity of numbness and tingling, health-related quality of life, and anxiety and depression, with effects sustained for at least six months post-treatment for several of these measures.

While these initial findings are encouraging, the evidence for O3T in CIPN is still emerging, and further research in this area is crucial to definitively establish the efficacy and safety of O3T. Our ongoing studies on CIPN aim to validate these preliminary results through RCTs, with one focused on pain (NCT04299893) and another focused on numbness and tingling (NCT06706544). Both are using (i) well-defined O3T protocols (route of administration, dose, frequency, and duration), and (ii) comprehensive and validated outcome measures, including PROs for pain, numbness, tingling, and functional impairment, with 3 months follow-up after the end of O3T to confirm the durability of the different effects and potential long-term adverse events.

Furthermore, our studies aim to evaluate several additional key objectives: (i) The role of O3T in modulating the gut–brain axis and its relevance to CIPN (NCT06799351). (ii) The molecular mechanisms associated with O3T effects in the context of CIPN, especially related to oxidative stress and inflammation (NCT04299893 and NCT06706544). (iii) The identification of predictive biomarkers of the response to O3T could help to develop personalized treatments and aid in the selection of patients who are most likely to benefit from therapy. This could include genetic, inflammatory, or oxidative stress markers. (iv) Finally, our RCTs are also exploring the potential role of hyperspectral imaging (HSI), a non-invasive and non-ionizing imaging modality that obtains spectral information from the captured tissue by analyzing a wide spectrum covering the visible (400–700 nm) to near-infrared (700–1000 nm) spectral ranges. We are exploring whether objective HSI evaluations are associated with subjective symptoms of CIPN and subsequent changes after O3T by calculating and correlating different tissue parameters, such as tissue oxygenation [88,89].

In conclusion, CIPN is a frequent and debilitating side effect of chemotherapy with limited therapeutic options. In preliminary studies, O3T has shown theoretical potential as an adjuvant therapy for managing CIPN symptoms, possibly targeting some of its underlying pathophysiological mechanisms. However, these findings should be interpreted cautiously due to the small sample sizes, lack of standardization, and variability across studies. Robust, well-designed RCTs are urgently needed to confirm the efficacy and safety of O3T. The next key steps include standardizing O3T protocols (e.g., dose, route, and duration) and validating reliable biomarkers, such as oxidative stress and inflammation markers, to better evaluate the therapeutic effects and identify patients who are most likely to benefit. If its efficacy is confirmed, O3T could become a valuable and well-tolerated tool to improve symptoms and quality of life for cancer survivors suffering from CIPN.

## Figures and Tables

**Table 1:** A summary of clinical studies and reviews on ozone therapy in the management of chemotherapy-induced peripheral neuropathy.

Study	Number of Patients	Experimental Design	Main Results
Clavo et al., Antioxidants 2019 [56].	(review)	Review of preclinical and limited clinical data on using ozone for chemotherapy toxicity	O3T may reduce chemotherapy-induced toxicity via antioxidant effects; limited clinical data for CIPN
Clavo et al., IJMS 2021 [11].	(review + trial protocol)	Review of mechanisms and clinical/experimental data; description of ongoing RCT	Suggests O3T may modulate oxidative stress/inflammation in CIPN; highlights need for RCTs
Clavo et al., Front Physiol 2022 [82].	7	Retrospective, preliminary report; rectal ozone insufflation in patients with chronic pain due to grade II/III CIPN; pain was assessed via VAS at baseline, end of treatment, and 3 and 6 months	Clinically relevant pain reduction in most patients; median VAS decreased from 7 to 4 at end of treatment; effect maintained at 3 and 6 months; CTCAE pain toxicity grade improved in 50%
Clavo et al., IJERPH 2023 [84].	26 (15 with CIPN)	Retrospective study; ozone in cancer survivors with chronic toxicity (radiotherapy/chemotherapy); HRQOL and toxicity assessed pre/post	Significant improvement in HRQOL and toxicity grade, including the subgroup of patients with CIPN
Szklener et al., IJMS 2023 [12]	(review)	Narrative review of ozone in CIPN	Summarizes rationale and limited clinical evidence; calls for RCTs
Clavo et al., Front Psychol. 2023 [85]	16 (8 with CIPN)	Retrospective study; several ozone treatments (mostly via rectal insufflation) in patients with refractory symptoms; assessment of anxiety and depression at baseline, post-treatment, and 3 and 6 months	Significant improvement in anxiety and depression measured via two questionnaires. Subgroup with CIPN not specified
Clavo et al., Integr Cancer Ther 2025 [83]	15	Retrospective study; rectal ozone insufflation (40 sessions over 4 months) in patients with persistent numbness/tingling due to grade II/III CIPN; assessment at baseline, post-treatment, and 3 and 6 months	47% showed reduction in CIPN toxicity grade (*p* = 0.016); 67% reported ≥50% reduction in numbness/tingling (*p* = 0.002); effects sustained at 3 and 6 months

**Note:** CIPN, chemotherapy-induced peripheral neuropathy; CTCAE, Common Terminology Criteria for Adverse Events; HRQOL, health-related quality of life; O3T, ozone therapy; RCT, randomized controlled trial; VAS, visual analog scale.

## Data Availability

Data supporting the reported results in this review can be found in the respective references.

## Abbreviations

The following abbreviations are used in this manuscript:

2,3-BPG	2,3-Bisiphosphoglycerate.
4-HNE	4-Hydroxynonenal.
ACTG1	Actin Gamma 1.
c-FOS	FBJ Murine Osteosarcoma Viral Oncogene Homolog.
AMPK	Adenosine 5′-Monophosphate-Activated Protein Kinase.
ASCO	American Society of Clinical Oncology.
BPG	Bisphosphoglycerate.
CAPG	Capping Actin Protein, Gelsolin-Like.
CAT	Catalase.
CaV	Voltage-Gated Calcium Channel.
CGRP	Calcitonin Gene-Related Peptide.
CIPN	Chemotherapy-Induced Peripheral Neuropathy.
CTCAEs	Common Terminology Criteria for Adverse Events.
CYP	Cytochrome P450.
DRG	Dorsal Root Ganglion.
eNOS	Endothelial Nitric Oxide Synthase.
EQ-5D-5L™	EuroQol-5 Dimension-5 Level Questionnaire.
EQ-VAS	Self-Perceived General Health Status.
GABA	Gamma-Aminobutyric Acid.
GPCRs	G-Coupled Protein Receptors.
GPx	Glutathione Peroxidase.
GSK3β	Glycogen Synthase Kinase 3 Beta.
GST	Glutathione S-Transferase.
GWAS	Genome-Wide Association Studies.
H2O2	Hydrogen Peroxide.
HADS	Hospital Anxiety and Depression Scale.
HIF-1α	Hypoxia-Inducible Factor-1α.
HO-1	Heme Oxygenase-1.
HRQOL	Health-Related Quality of Life.
HSI	Hyperspectral Imaging.
HSP70	Heat Shock Protein 70.
IL	Interleukin.
iNOS	Inducible Nitric Oxide Synthase.
KV	Voltage-Gated Potassium Channel.
LOPs	Lipid Oxidation Products.
MAHT	Major Autohemotherapy.
MAPK	Mitogen-Activated Protein Kinase.
MAPT	Microtubule-Associated Protein Tau.
MMP	Matrix Metalloproteinase.
MRI	Magnetic Resonance Imaging.
NaV	Voltage-Gated Sodium Channel.
NF-κB	Nuclear Factor Kappa B.
NK	Natural Killer.
Nrf2	Nuclear Factor Erythroid 2-Related Factor 2.
O3T	Ozone Treatment.
PROs	Patient-Reported Outcomes.
RCT	Randomized Controlled Trial.
ROS	Reactive Oxygen Species.
SNPs	Single-Nucleotide Polymorphisms.
SOCS3	Suppressors Of Cytokine Signaling 3.
SOD	Superoxide Dismutase.
TF	Tissue Factor.
TGF-β1	Transforming Growth Factor Beta 1.
TLR4	Toll-Like Receptor 4.
TNF-α	Tumor Necrosis Factor-Alpha.
TRP	Transient Receptor Potential.
TUBB2A	Tubulin Beta 2A Class IIa.
VAS	Visual Analog Scale.
